# Deep learning approximation of attenuation maps for myocardial perfusion SPECT with an IQ$\varvec{\cdot {}}$SPECT collimator

**DOI:** 10.1186/s40658-023-00568-1

**Published:** 2023-08-28

**Authors:** Tamino Huxohl, Gopesh Patel, Reinhard Zabel, Wolfgang Burchert

**Affiliations:** 1 Institute of Radiology, Nuclear Medicine and Molecular Imaging, Heart and Diabetes Center North Rhine-Westphalia, University Hospital of the Ruhr University Bochum, Bad Oeynhausen, Germany; 2 Institute of Nuclear Medicine, Hospital Lippe, Lippe, Germany

**Keywords:** Deep learning, SPECT, Myocardial perfusion imaging, Attenuation correction

## Abstract

**Background:**

The use of CT images for attenuation correction of myocardial perfusion imaging with single photon emission computer tomography (SPECT) increases diagnostic confidence. However, acquiring a CT image registered to a SPECT image is often not possible because most scanners are SPECT-only. It is possible to approximate attenuation maps using deep learning, but this has not yet been shown to work for a SPECT scanner with an IQ$\varvec{\cdot {}}$SPECT collimator. This study investigates whether it is possible to approximate attenuation maps from non-attenuation-corrected (nAC) reconstructions acquired with a SPECT scanner equipped with an IQ$\varvec{\cdot {}}$SPECT collimator.

**Methods:**

Attenuation maps and reconstructions were acquired retrospectively for 150 studies. A U–Net was trained to predict attenuation maps from nAC reconstructions using the conditional generative adversarial network framework. Predicted attenuation maps are compared to real attenuation maps using the normalized mean absolute error (NMAE). Attenuation-corrected reconstructions were computed, and the resulting polar maps were compared by pixel and by average perfusion per segment using the absolute percent error (APE). The training and evaluation code is available at https://gitlab.ub.uni-bielefeld.de/thuxohl/mu-map.

**Results:**

Predicted attenuation maps are similar to real attenuation maps, achieving an NMAE of 0.020±0.007. The same is true for polar maps generated from reconstructions with predicted attenuation maps compared to CT-based attenuation maps. Their pixel-wise absolute distance is 3.095±3.199, and the segment-wise APE is 1.155±0.769.

**Conclusions:**

It is feasible to approximate attenuation maps from nAC reconstructions acquired by a scanner with an IQ$\varvec{\cdot {}}$SPECT collimator using deep learning.

## Background

Single photon emission computer tomography (SPECT) myocardial perfusion imaging (MPI) is a widely used technique for the assessment of coronary artery disease (CAD). Unfortunately, photon attenuation severely limits the interpretability of the images for this purpose [1, 2]. Therefore, non-diagnostic CT images are acquired with dedicated SPECT/CT scanners that can be converted into attenuation coefficients. The resulting attenuation maps are used for attenuation-corrected (AC) reconstruction.

However, there are a number of drawbacks and barriers to this approach. First and foremost, SPECT/CT scanners are more expensive than SPECT-only scanners, resulting in less widespread availability. In fact, the market share of SPECT systems that include a CT for AC is only 20% [3]. Another problem is that although it is non-diagnostic, low-dose CT, it still exposes the patient to additional radiation. In terms of patient health, it would be better to either avoid this radiation or use it for other diagnostic tests [4]. However, it has not yet been conclusively determined whether non-diagnostic CT can still provide useful information, e.g., for the estimation of coronary calcification [5]. Finally, there is the problem of patient motion, which can be a source of misregistration between SPECT and CT scans. Misregistration can lead to artifacts in the reconstructed images [6, 7].

**Table 1:** Overview of existing research on AC of MPI SPECT with deep learning.

Paper	Scanner (Manufacturer)	Collimator	Method	Scatter window
[8]	Discovery NM/CT 570c (GE)	Pinhole	Direct	Yes
[9]	Discovery NM/CT 570c (GE)	Pinhole	Both	Yes
[9]	NM/CT 850c (GE)	LEHR	Both	Yes
[10]	NM/CT 850 (GE)	LEHR	Indirect	Yes
[11]	Unknown	–	Direct	No
[12]	Discovery NM/CT 570c (GE)	Pinhole	Direct	No
[13]	Discovery NM/CT 670 (GE)	LEHR	Direct	Yes
[14]	NM/CT 850 (GE)	LEHR	Indirect	Yes
[14]	Brightview XCT (Philips)	LEHR	Indirect	Yes
[15]	Optima NM/CT 640 (GE)	LEHR	Indirect	No
[16]	Discovery NM/CT 570c (GE)	Pinhole	Direct	No
[16]	Discovery NM/CT 530c (GE)	Pinhole	Direct	No
[17]	Discovery NM/CT 570c (GE)	Pinhole	Direct	No
Ours	Symbia Intevo (Siemens)	IQ$\varvec{\cdot {}}$SPECT	Indirect	No

Note that the collimator is often not specified in the corresponding paper. In these cases, the European Association of Nuclear Medicine (EANM) recommendation is given [18, 19]

To address these issues, a current research goal is to approximate attenuation maps from non-attenuation-corrected (nAC) reconstructions using deep learning. A number of papers that have been published on this topic are listed in Table 1. They can be divided into direct and indirect approaches. The direct approach is to use a deep learning model to transform a nAC reconstruction into an AC reconstruction. In contrast, the indirect approach is to approximate an attenuation map from the nAC reconstruction, which in turn can be used to compute an AC reconstruction with the projection data. The direct approach is usually applied to dedicated cardiac SPECT scanners because they have a limited field of view (FOV) that does not cover the entire human body [8]. Recently, however, an indirect approach for dedicated cardiac SPECT scanners has been presented and found to yield better results than a direct approach [9]. Most of the previous studies, whether direct or indirect, make use of a reconstruction derived from counts of one or more lower energy windows (scatter windows) in addition to the normal reconstruction. These lower energy windows may contain useful information for approximating the attenuation map, and their inclusion has been shown to lead to better results [10].

**Fig. 1:**
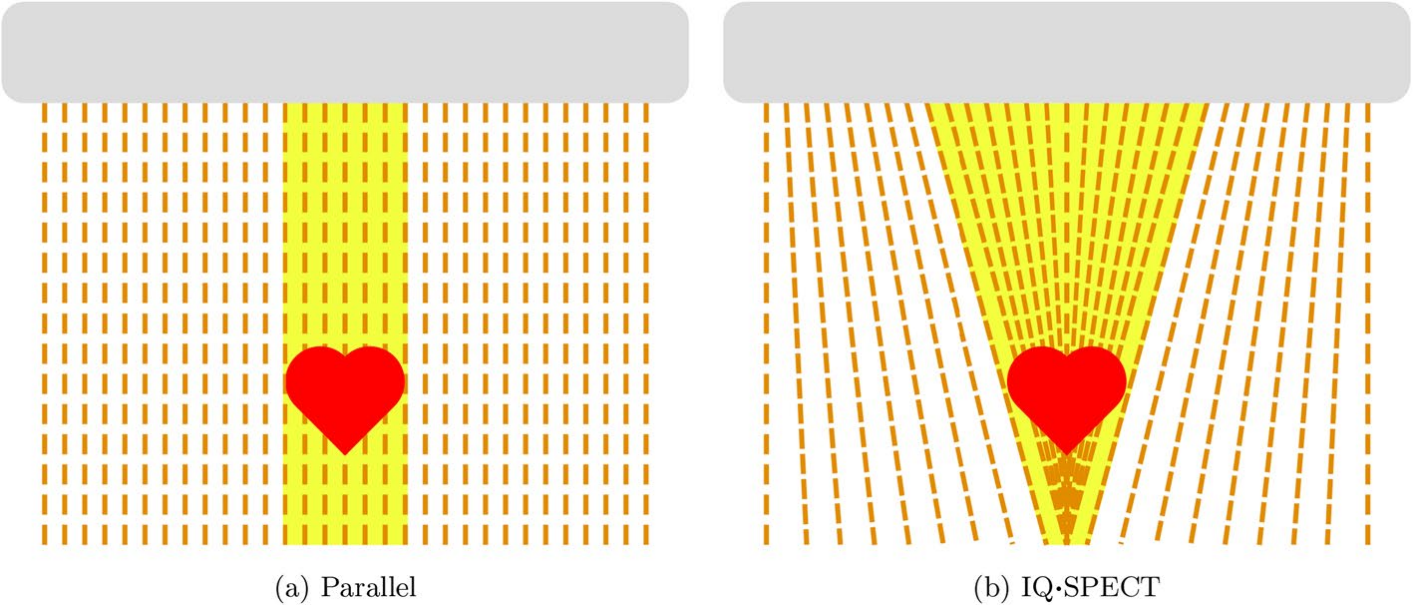
Different collimator designs. The illustration is inspired by [20]

In this study, we investigate whether the approximation of attenuation maps using deep learning works for scans from a Symbia Intevo SPECT/CT scanner (Siemens Healthineers, Erlangen, Germany). In contrast to previous studies, this scanner uses the IQ$\varvec{\cdot {}}$SPECT technology [21]. This means that it uses a multifocal collimator that focuses at its center and becomes near parallel at its edges. This design is illustrated in comparison to a conventional parallel collimator in Fig. 1. An additional difference is that the detector heads perform a cardio-centric rotation. As a result, it is a scanner with a FOV that covers the entire human body but still focuses on the heart. This design must be taken into account, especially during reconstruction. Verification with this scanner is important because deep learning performance is known to degrade with data not seen during training. Therefore, a clinical application requires a verification with as many modalities as possible. Note that there is already a study investigating AC for an IQ$\varvec{\cdot {}}$SPECT scanner [22]. However, this study differs from all the others in that the polar maps, not reconstructions, are transformed from nAC to AC.

Here, we implement the indirect approach without a scatter-window reconstruction. The indirect approach is used because the FOV of the scanner covers the entire human body, and as mentioned above, has been shown to produce better results. Furthermore, AC reconstruction with a slightly inaccurate attenuation map of the thorax can still produce an accurate reconstruction [23]. Therefore, this approach has a built-in fault tolerance against errors made by the deep learning model. In addition, the attenuation map is an intermediate result that can be reviewed by a physician for quality assurance. This could also help address the distrust of deep learning due to its black-box nature. The reason for not using a scatter window reconstruction is practical. The manufacturer’s software simply does not allow a reconstruction to be computed, even though scatter counts are acquired for scatter correction. Custom reconstruction is not possible for data acquired with the IQ$\varvec{\cdot {}}$SPECT setup. As a result, we argue that a deep learning approach to AC that aims to work with as many scanners as possible should not require scatter-window reconstructions, but should make their inclusion optional.

## Materials and methods

### Patients and image acquisition

For this retrospective study, 150 SPECT MPI scans from 103 patients were collected from July 2021 to May 2022. The data include stress, rest, stress/rest and rest/stress studies, which explains why there are more scans than patients. All scans were acquired on a Symbia Intevo SPECT/CT scanner with patients in the supine position. In the case of stress/rest and rest/stress studies, a separate CT was acquired for each protocol. Data were split by patient into training/validation/test sets in a 70:15:15 ratio. Clinical characteristics of patients by split are shown in Table 2.

**Table 2:** Gender, age, height and weight of the patients and the imaging protocol in the data by split. M indicates male, F female, S stress and R rest. For the numerical values, the mean, the standard deviation and the value range are given

Split	Gender	Age (year)	Height (cm)	Weight (kg)	BMI	Protocol
Training	72 M, 20 F	66.0±11.0	174.1±9.2	86.1±18.7	28.3±5.0	76 S, 34 R
		34 to 88	158 to 195	48 to 151	18.5 to 40.7	
Validation	11 M, 5 F	66.2±9.1	171.1±6.7	88.3±19.0	30.0±5.6	14 S, 5 R
		49 to 84	158 to 180	60 to 129	22.6 to 45.2	
Test	8 M, 7 F	68.1±9.5	172.0±9.5	91.5±23.2	31.1±7.8	15 S, 6 R
		40 to 83	155 to 186	57 to 133	18.6 to 46.3	

SPECT projection data were acquired with two detector heads covering a 208$^{\circ }$ orbit with a step size of 6$^{\circ }$, corresponding to 34 angles. Counts were accumulated for a photopeak energy window of 129 to 150 keV. While counts were also accumulated for a scatter energy window of 108 to 129 keV, they could not be reconstructed, but could only be used for scatter correction with the scanner software. For this study, reconstruction with and without scatter correction was performed using the ordered subset expectation maximization (OSEM) algorithm as implemented in the manufacturer’s software with 10 iterations and 3 subsets. The triple energy window method was used for scatter correction [24]. Reconstructions have a resolution of 128$\times$ 128$\times$128 voxels with a voxel size of 4.8$\times$ 4.8$\times$4.8 ${\textrm{mm}}^{3}$. CTs were acquired with a variable number of slices in the axial direction ranging from 23 to 82. They were manually aligned to the nAC reconstructions and converted to linear attenuation coefficients in units of ${\textrm{cm}}^{-1}$ using the scanner software. Subsequently, AC reconstructions were computed using the same OSEM algorithm, and both nAC and AC reconstructions were cropped to the number of CT slices.

### Deep learning model

**Fig. 2:**
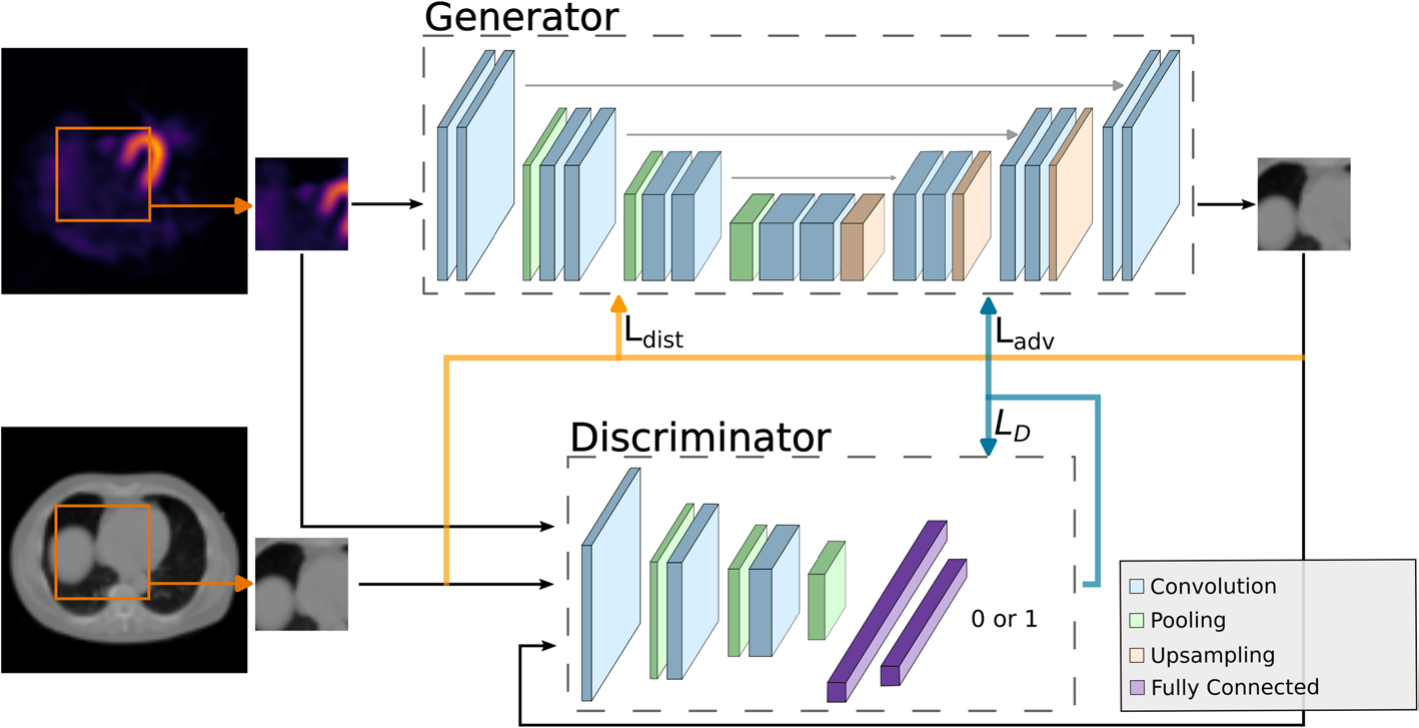
Illustration of the cGAN training procedure

To transform nAC reconstructions into attenuation maps, we use the conditional generative adversarial network (cGAN) framework for image-to-image translation [25]. In this framework, two models are trained by competing with each other: the generator transforms a reconstruction into an attenuation map, while the discriminator tries to discriminate whether a pair of a reconstruction and attenuation map is real or fake (generated by the generator). It has been shown that this framework leads to better results than simply optimizing the distance between a real and a predicted image, not only for attenuation map generation [10], but also for RGB image generation [26]. Figure 2 shows the cGAN framework for predicting attenuation maps.

Regarding model architectures, we follow [10] and use a 3D U–Net [27] as a generator. The U–Net architecture is well suited for tasks where the input and output are structurally similar, since low-level structural information has a shortcut to the output. As in [10], we add batch normalization to each convolutional layer and dropout of 0.15 to the bottleneck layer. We test different depths for the generator: either four levels deep, starting with 64 filters in the first level, or five levels deep, starting with 32 filters. In both cases, the number of filters doubles with each level.

As a discriminator, we use a typical convolutional neural network for image classification or a PatchGAN [25]. Both consist of a few levels of convolutional layers and max pooling. The difference between the two is that the classification network ends with fully connected layers, allowing it to discriminate whole images, while the PatchGAN uses convolutional layers in a way that it discriminates between overlapping image patches. Similar to the generator, we test different model depths and numbers of convolutional filters.

In the cGAN framework with discriminator *D* and generator *G*, the discriminator is trained with the loss

1 $$L_{D}(X, Y) =0.5{\Bigl (D\bigl (X, Y\bigl ) - 1\Bigl )}^{2} + 0.5{\Bigl (D\bigl (X, G(X)\bigl ) - 0\Bigl )}^{2},$$

where *X* is the SPECT image, *Y* is the target attenuation map, and *G*(*X*) is the attenuation map generated by the generator. The generator is trained using the loss

2 $$L_{G}(X, Y) =\lambda _{\textrm{adv}}{} L_{\textrm{adv}}{}\bigl (X, Y\bigl ) + \lambda _{\textrm{dist}}{} L_{\textrm{dist}}{}\bigl (G(X), Y\bigl ),$$

where $L_{\textrm{adv}}{}(X, Y)$ is the adversarial loss, which is the inverted discriminator loss so that fooling it is optimized, and $L_{\textrm{dist}}{}(G(X), Y)$ is a loss that evaluates the similarity between the generated attenuation map and the real one. The parameters $\lambda _{\textrm{adv}}$ and $\lambda _{\textrm{dist}}$ are weights for the respective losses. Common implementations of $L_{\textrm{dist}}$ are the mean absolute error ($L_{1}$), the mean squared error ($L_{2}$), or a combination of $L_{2}$ and the gradient difference loss $L_{\textrm{GDL}}$ [28].

### Image pre-processing and training parameter optimization

Again, according to [10], the patient bed is manually cropped from the target attenuation map and reinserted before evaluation. Bed removal and reinsertion can also be automated by applying a threshold to the attenuation map and selecting the lowest region as the patient bed. Furthermore, reconstructions and attenuation maps are zero-padded to a minimum size of 32 slices. Afterward, patches with a resolution of either 32$\times$ 32$\times$32 or 32$\times$ 64$\times$64 are randomly sampled for model training. For the smaller resolution, 50 to 100 patches were sampled from each image, while for the larger resolution, 25 to 50 patches were sampled to keep the training time similar. Another important pre-processing step is image normalization, of which there are two common types:

1. Mean: The SPECT image is divided by its mean value.
2. Gaussian: The mean value is subtracted from the SPECT image and it is divided by its standard deviation.

All of the parameters mentioned so far (model depths, number of convolutional filters, type of discriminator, type of distance loss, loss weights $\lambda _{\textrm{adv}}$ and $\lambda _{\textrm{dist}}$, patch size, number of patches, type of normalization and reconstruction with or without scatter correction) are optimized by random search [29]. In random search, model training is repeated for many iterations, and all parameters are randomly sampled before each iteration. This is usually faster than grid search because less time is wasted on irrelevant parameters. In each iteration, a model is trained for 100 epochs with a learning rate of 0.001 and a batch size of 64 for small patches and 32 for large patches. For each iteration, the model weights of the epoch with the minimum $L_{1}$ loss for the validation split were stored. A total of 215 random search iterations were performed.

### Data analysis

To analyze the random search, the normalized mean absolute error (NMAE) between the generated attenuation map and the real attenuation map is calculated for each image in the validation split of the data. Based on the average NMAE, a statistical analysis is used to evaluate which hyperparameters lead to a significant improvement. To do this, the Shapiro-Wilk test is used to determine whether the distribution of scores for each parameter choice is normal, and if so, it is compared with other choices using the t test. The average NMAE is also used to select the best performing model. Note that about one fourth of the random search iterations (50 out of 215) were discarded because the training did not converge to predict adequate attenuation maps. These iterations were manually sorted out by looking at a few generated attenuation maps for the validation split.

For the best model, attenuation maps are produced for the test split and the NMAE is calculated. In addition, the attenuation maps are used to compute AC reconstructions using a custom implementation of the post-reconstruction attenuation correction (PRAC) algorithm [30]. The PRAC algorithm is used because we could not use the generated attenuation maps in the scanner software and because it is independent of the scanner used. The custom implementation of PRAC uses the open source reconstruction software STIR [31] and its extension to SPECT [32]. The AC reconstructions are used to generate polar maps using the Cedars-Sinai Cardiac Suite [33]. Polar maps are compared pixel-by-pixel using the APE and by evaluating their correlation. The same is done for the average perfusion per segment of the polar maps.

## Results

In total, it took nearly two and a half weeks to run 215 iterations of random search on a single computer with an NVIDIA RTX A6000 graphics card. On average, it takes just over two hours to train a single model. The following presentation of random search results is limited to those that show a significant improvement based on the t test.

**Fig. 3:**
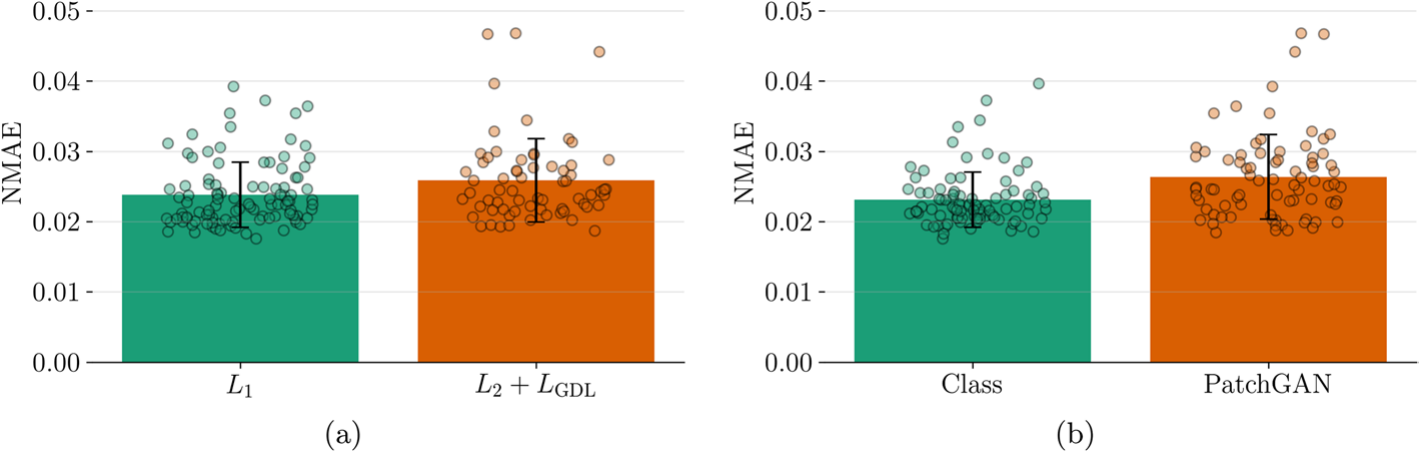
Training results depending on the type of distance loss and the type of discriminator. The bars show the average NMAE and individual results are scattered

These are the type of distance loss (Fig. 3a) and the type of discriminator (Fig. 3b). Using the $L_{1}$ loss (0.024±0.005) as the distance loss is significantly better p=0.01 than using $L_{2} + L_{\textrm{GDL}}{}$ (0.026±0.006). Using a classification neural network as a discriminator (0.023±0.004) is significantly better (p= 0.00006) than using a PatchGAN (0.02636±0.006).

In contrast to [10], using mean normalization (0.025±0.006) is not more robust than Gaussian normalization (0.024±0.005) in our experiment. However, the reason for this may be that non-convergent iterations were discarded. While only 12 out of 72 iterations trained with mean normalization were discarded, it was 20 out of 72 for Gaussian normalization. Other factors that have a strong influence on training convergence are the loss weights $\lambda _{\textrm{adv}}$ and $\lambda _{\textrm{dist}}$. Choosing $\lambda _{\textrm{adv}}$ larger than $\lambda _{\textrm{dist}}$ increases the likelihood that the training will not converge. While only 8 out of 34 iterations with $\lambda _{\textrm{adv}}$=1, $\lambda _{\textrm{dist}}$=100 did not converge, it was 11 out of 34 for $\lambda _{\textrm{adv}}$=100, $\lambda _{\textrm{dist}}$=1.

**Fig. 4:**
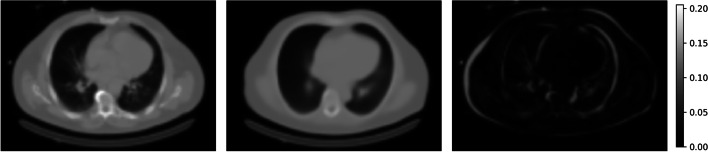
From left to right: an attenuation map slice, the corresponding best model prediction and the absolute difference between the two slices. The color bar on the right encodes the attenuation in cm$^{-1}$

The best iteration achieved an NMAE of 0.018±0.006 on the validation split and 0.020±0.007 on the test split. Training was performed using patches with a resolution of 64$\times$ 64$\times$64 voxels, mean normalization and loss weights $\lambda _{\textrm{adv}}$=1 and $\lambda _{\textrm{dist}}$=100. The generator is a four-level deep U–Net with 64, 128, 256 and 512 convolutional filters per level. The discriminator is a typical image classification network with three levels of convolution with 64, 128 and 256 filters, followed by two fully connected layers with 512 and 128 neurons. Training reached the minimum validation loss after 60 epochs. A prediction of an attenuation map by this model is shown next to a real attenuation map in Fig. 4 to give an idea of the quality of the predicted attenuation maps.

**Fig. 5:**
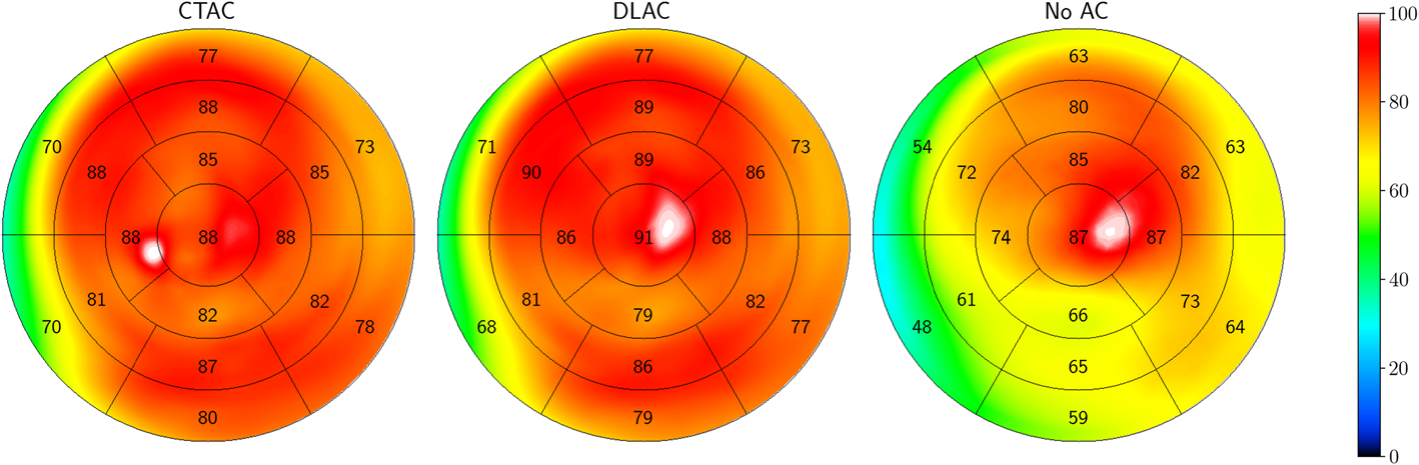
An example of the polar maps generated with CTAC, DLAC and nAC reconstructions. Numbers and color indicate the average perfusion per segment

**Fig. 6:**
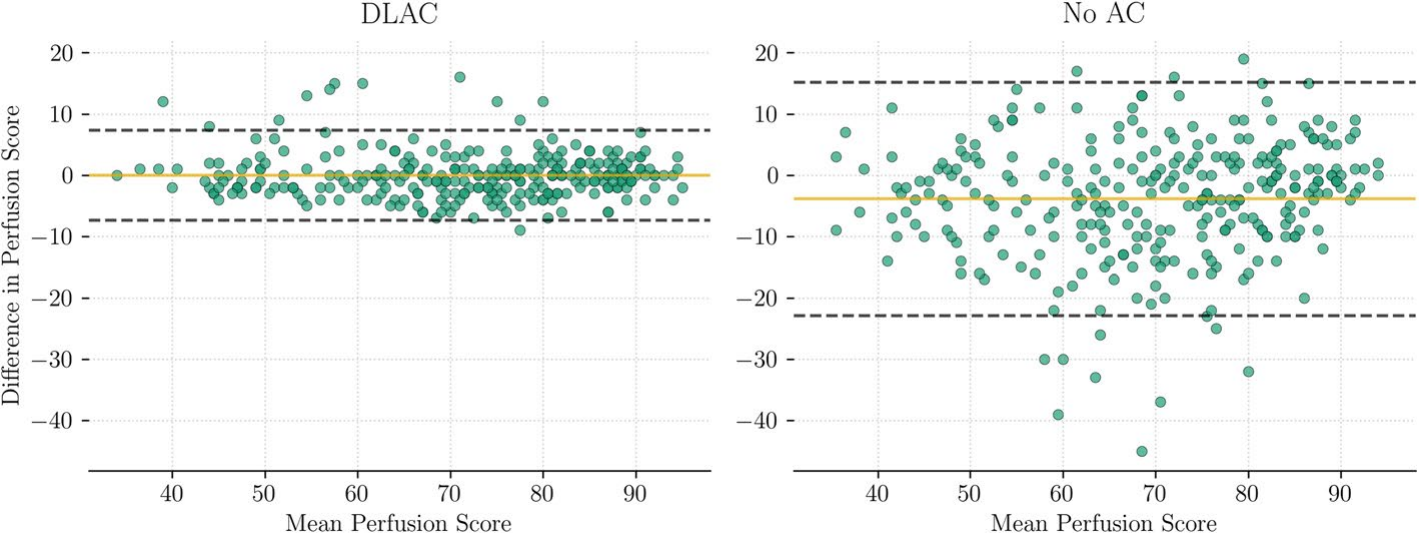
Bland–Altman plots for the 17-segment perfusion scores of the test split images. They show the difference between DLAC and CTAC on the left and nAC and CTAC on the right

**Fig. 7:**
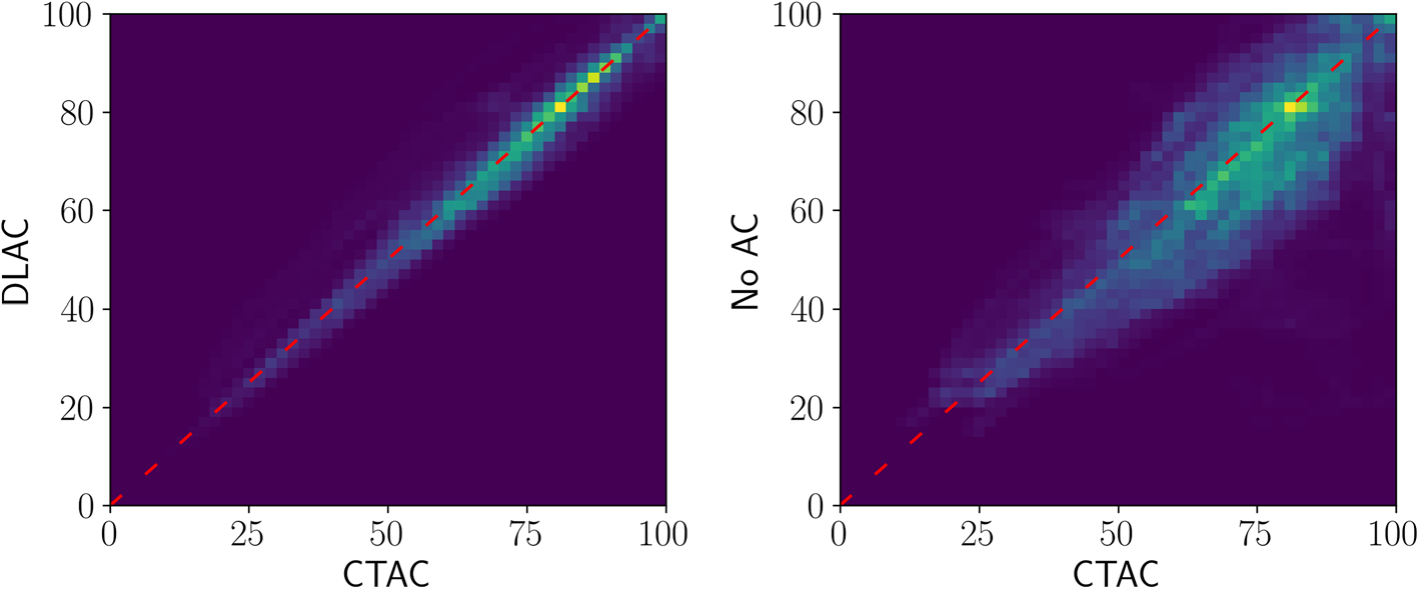
Pixel-wise correlation maps between DLAC and CTAC polar maps on the left and nAC and CTAC polar maps on the right for all test split images. The correlation was calculated using pixels in the circular region of the polar maps at a resolution of $512\times {}512$

Figure 5 shows an example of polar maps generated using CT-based AC (CTAC), deep learning-based AC (DLAC) and nAC reconstructions. Note that both the CTAC and the DLAC reconstructions were computed using the PRAC algorithm. In this example, an improvement due to attenuation correction is clearly visible, and DLAC produces a similar polar map to CTAC. This qualitative observation is further supported by a quantitative evaluation of the perfusion scores and pixels of the polar maps. For this purpose, Fig. 6 shows Bland–Altman plots for the 17-segment perfusion scores, and Fig. 7 shows pixel-wise correlation maps. Both show a high agreement between CTAC and DLAC, while nAC often underestimates perfusion. The APE of the average perfusion per segment between DLAC and CTAC is 1.155±0.769, while it is 7.26428±5.48144 for nAC. The correlation coefficient is R=0.97 for DLAC and R=0.79 for nAC. Similarly, the absolute pixel distance is 3.095±3.199 with a correlation coefficient of R=0.97 for DLAC and 8.559±7.665 with a correlation coefficient of R=0.81 for nAC.

## Discussion

In this work, it was tested whether it is possible to approximate attenuation maps for reconstructions acquired with a SPECT scanner equipped with an IQ$\varvec{\cdot {}}$SPECT collimator using deep learning. For this purpose, a U–Net was trained using the cGAN framework. The influence of training parameters was investigated using a random search procedure. It was shown that using an $L_{1}$ loss as the distance loss is preferable to $L_{2} + L_{\textrm{GDL}}{}$. Furthermore, using a classification network as a discriminator gave better results than using a PatchGAN, and cGAN training converged more reliably when the distance loss was weighted higher than the adversarial loss. Other training parameters, such as the use of reconstructions with or without scatter correction, did not significantly affect the results.

The best model trained by the random search procedure produces attenuation maps that are similar to the real attenuation maps. Predicted and actual attenuation maps differ only in the fine structure and at the body edges (see Figure 4). That the fine structure cannot be recovered is to be expected, since it does not contribute significantly to the distance loss used to train the generator. The reason for the differences at the body edges could be that SPECT images are acquired at a lower resolution than CT images, which means that the edges cannot be recovered as well. However, both differences are small overall, which means that they do not considerably affect the use of the predicted attenuation maps for attenuation correction. This is shown by computing AC reconstructions with the PRAC algorithm and subsequently generating polar maps. Compared to nAC polar maps, DLAC polar maps have a high pixel-wise and average perfusion per segment agreement with CTAC polar maps.

A limitation of this study in terms of practical application is that AC reconstructions and polar maps were not tested to determine whether diagnostically relevant information is lost. The measures used for comparison assess similarity but do not necessarily account for diagnostically relevant features. Besides, the manual handling of the patient bed hinders the practical application. While it is certainly possible to measure the patient bed and insert it into the attenuation map predicted by the deep learning model, a more elegant solution, which should be investigated in future work, is for the model to estimate the bed directly from the emission data, such as shown in [9]. In addition, we note that there is a difference between the CTAC reconstructions computed with the PRAC algorithm and the CTAC reconstruction computed with the vendor software. Compared to the vendor reconstructions, the DLAC reconstructions computed with the PRAC algorithm are still more similar than the nAC reconstructions, but they are less close. However, a comparison between CTAC and DLAC reconstructions computed with the PRAC should be a better indicator of the suitability of the predicted attenuation map for reconstruction, since the reconstruction algorithm is the same while only the input is different. Finally, the results need to be validated with an inter-institutional dataset and it needs to be tested if a model can be trained that can handle multiple scanners simultaneously.

## Conclusion

This paper shows that deep learning can be used to approximate attenuation maps from nAC reconstructions acquired with a scanner equipped with an IQ$\varvec{\cdot {}}$SPECT collimator. The approximation is possible even without the use of scatter window reconstructions, which could not be obtained from the manufacturer’s software. Furthermore, it is shown that an $L_{1}$ distance loss and a classification network as a discriminator are the best choices for training a U–Net with the cGAN framework for this purpose.

## Data Availability

The datasets generated and analyzed in the current study are not publicly available, as the patients did not consent to publicly share their data. However, they are available in a pseudonymized format from the corresponding author on reasonable request. Code is available in the repository https://gitlab.ub.uni-bielefeld.de/thuxohl/mu-map.
